# Factors associated with mortality in hospitalized cardiovascular disease patients infected with COVID‐19

**DOI:** 10.1002/iid3.561

**Published:** 2022-01-20

**Authors:** Roohallah Alizadehsani, Rahimeh Eskandarian, Mohaddeseh Behjati, Mehrdad Zahmatkesh, Mohamad Roshanzamir, Navid H. Izadi, Afshin Shoeibi, Azadeh Haddadi, Fahime Khozeimeh, Fariba A. Sani, Zahra A. Sani, Zahra Roshanzamir, Abbas Khosravi, Saeid Nahavandi, Nizal Sarrafzadegan, Sheikh Mohammed Shariful Islam

**Affiliations:** ^1^ Institute for Intelligent Systems Research and Innovation (IISRI) Deakin University Geelong Australia; ^2^ Internal Medicine Research Center Semnan University of Medical Sciences Semnan Iran; ^3^ Rajaei Cardiovascular Medical and Research Center Iran University of Medical Sciences Tehran Iran; ^4^ Department of Engineering Fasa University Fasa Fars Iran; ^5^ Department of Electrical and Computer Engineering Isfahan University of Technology Isfahan Iran; ^6^ Computer Engineering Department Ferdowsi University of Mashhad Mashhad Iran; ^7^ Faculty of Electrical and Computer Engineering, Biomedical Data Acquisition Lab K. N. Toosi University of Technology Tehran Iran; ^8^ Department of Biology, Faculy of Basic Sciences, Shahrekord Branch Islamic Azad University Shahrekord Iran; ^9^ Faculty of Medicine Mashhad University of Medical Science Mashhad Iran; ^10^ Department of Cardiac MRI Omid Hospital Tehran Iran; ^11^ Pediatric Respiratory and Sleep Medicine Research Center, Children's Medical Center Tehran University of Medical Sciences Tehran Iran; ^12^ Isfahan Cardiovascular Research Center, Cardiovascular Research Institute Isfahan University of Medical Sciences Isfahan Iran; ^13^ Faculty of Medicine, SPPH University of British Columbia Vancouver British Columbia Canada; ^14^ Institute for Physical Activity and Nutrition, School of Exercise and Nutrition Sciences Deakin University Geelong Victoria Australia; ^15^ Cardiovascular Division The George Institute for Global Health Newtown Australia; ^16^ Sydney Medical School University of Sydney Camperdown Australia

**Keywords:** cardiovascular patients, COVID‐19, mortality, signs and symptoms

## Abstract

**Introduction:**

To reduce mortality in hospitalized patients with COVID‐19 and cardiovascular disease (CVD), it is necessary to understand the relationship between patient's symptoms, risk factors, and comorbidities with their mortality rate. To the best of our knowledge, this paper is the first which take into account the determinants like risk factors, symptoms, and comorbidities leading to mortality in CVD patients who are hospitalized with COVID‐19.

**Methods:**

This study was conducted on 660 hospitalized patients with CVD and COVID‐19 recruited between January 2020 and January 2021 in Iran. All patients were diagnosed with the previous history of CVD like angina, myocardial infarction, heart failure, cardiomyopathy, abnormal heart rhythms, and congenital heart disease before they were hospitalized for COVID‐19. We collected data on patient's signs and symptoms, clinical and paraclinical examinations, and any underlying comorbidities. *t* test was used to determine the significant difference between the two deceased and alive groups. In addition, the relation between pairs of symptoms and pairs of comorbidities has been determined via correlation computation.

**Results:**

Our findings suggest that signs and symptoms such as fever, cough, myalgia, chest pain, chills, abdominal pain, nausea, vomiting, diarrhea, and anorexia had no impact on patients' mortality. There was a significant correlation between COVID‐19 cardiovascular patients' mortality rate and symptoms such as headache, loss of consciousness (LOC), oxygen saturation less than 93%, and need for mechanical ventilation.

**Conclusions:**

Our results might help physicians identify early symptoms, comorbidities, and risk factors related to mortality in CVD patients hospitalized for COVID‐19.

## Graphical abstract

Our findings suggest that signs and symptoms such as fever, cough, myalgia, chest pain, chills, abdominal pain, nausea, vomiting, diarrhea, and anorexia had no impact on patients` mortality. There was a significant correlation between COVID‐19 cardiovascular patients' mortality rate and symptoms such as headache, loss of consciousness (LOC), oxygen saturation less than 93%, and need for mechanical ventilation. Our results might help physicians identify early symptoms, comorbidities, and risk factors related to mortality in cardiovascular disease patients hospitalized for COVID‐19.

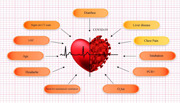

## 1 INTRODUCTION

Since the first case of coronavirus disease 2019 (COVID‐19) reported in December 2019, substantial mortality and morbidity have occurred globally due to this virus.^1^, ^2^ The World Health Organization declared COVID‐19 a pandemic on March 11, 2020, and appealed to all the countries to take aggressive actions to contain the spread of this disease.^2^, ^3^, ^4^ Patients who have underlying comorbidities, as well as elderly persons, are at high risk of COVID‐19 severity and mortality.^5^, ^6^ Studies indicated that severe and mortality cases are more likely associated with preexisting medical conditions, including but not limited to cardiovascular disease (CVD), cancer, cerebrovascular diseases, chronic obstructive pulmonary disease (COPD), diabetes, hypertension, and digestive diseases.^7^ CVD is a common comorbidity among patients with COVID‐19.^8^ Few earlier studies had ascertained that CVD as underlying comorbidity might enhance the risk of mortality in COVID‐19 patients.^9^, ^10^ Ssentongo studied 11 comorbidities such as CVD, hypertension, and diabetes and concluded that these comorbidities have direct impact on increased risk of mortality due to COVID‐19.^11^ In a similar study, the impact of CVD, hypertension, and myocardial injury was investigated.^12^ A recent study demonstrated that diabetic COVID‐19 patients without other comorbidities are at higher risk for excessive inflammation responses, the release of tissue injury‐related enzymes, severe pneumonia, and death.^13^ Studies yielded that cardiac injury, manifested by cardiac biomarker elevation, is identified in a considerable percentage of COVID‐19 patients and is associated with unfavorable outcomes and enhanced mortality.^14^ Several studies have investigated the impact of different diseases on the mortality rate of COVID‐19 patients. Rastad et al.^5^ investigated the predictors and risk factors of COVID‐19 patients with CVD and diabetes mellitus (DM) and in‐hospital mortality. A multivariate logistic regression was used to predict the risk of in‐hospital death for COVID‐19 patients with comorbidities like DM, CVD, and cancer. In their study, CVD patients yielded nonsignificant associations with the risk of death from COVID‐19. The authors reported lymphocyte count, creatinine, and C‐reactive protein (CRP) level as significant predictors of mortality due to COVID‐19 for patients with DM. Gu et al.^7^ assessed the impact of preexisting comorbidities on the hazard of COVID‐19 in mainland China by using a nested case‐control design. They utilized inverse probability‐weighted Cox proportional hazard model to explore the death risk of comorbidities of interest. Li et al.^8^ aimed to determine the clinical outcome and observations of COVID‐19 patients with and without CVD. Their multivariable Cox regression models showcased that CVD and older age were independent risk factors for mortality. In another study, Mehra et al. have also inspected the factors contributing to in‐hospital death of COVID‐19 patients.^15^ They reported that age above 65, having coronary artery disease (CAD), heart failure, cardiac arrhythmia, COPD, and smoking are among the key factors contributing to in‐hospital death. The authors have also reported that angiotensin‐converting–enzyme (ACE) inhibitors and angiotensin‐receptor blockers (ARBs) have no effect on increasing the risk of in‐hospital death.

Elderly patients are usually associated with higher risk of mortality. However, COVID‐19 is considered a threat for younger adults as well. Thus, Hendren et al.^16^ have investigated the contribution of obesity to in‐hospital death of COVID‐19 patients with special focus on younger adults (age ≤ 50). To this end, body mass index (BMI) was stratified according to the World Health Organization obesity class. Overall, the authors reported that obese patients are at higher risk of mortality due to COVID‐19.

Qin et al.^17^ studied the prognostic power and associations of poor outcomes of COVID‐19 with circulating cardiac injury markers. Li et al.^18^ studied the COVID‐19 patients admitted to Tongji Hospital, China, and evaluated the severity on admission, treatment, complications, and their outcomes. They executed survival analysis in severe patients using Cox proportional hazard regression model. Due to the importance of assessing characteristics of the COVID‐19 virus, Aggarwal et al.^19^ have reviewed 18 studies (two studies from the United States and 16 studies from China) to investigate the association of CVD with COVID‐19. The authors reported that preexisting CVD significantly increases the risk of severe form of COVID‐19 as well as the risk of mortality. Another study focused on Danish citizens with CVD.^20^ The study was conducted before and during lockdown in 2019 and 2020. The authors reported lower in‐hospital mortality rate and higher out‐of‐hospital mortality rate during lockdown compared to before lockdown. The obtained results were reported to be independent of age and sex.

Although clinical and epidemiological characteristics of COVID‐19 patients have been studied, a few studies have described the clinical course of ailment and risk factors for mortality. Zhou et al.^18^ included 191 patients, 91 of which had comorbidity, with hypertension (30% of patients), diabetes (19% of patients), and CAD (8% of patients). They applied multivariable and univariable logistic regression techniques to exploit the risk factors associated with in‐hospital death. Sabatino et al.^21^ evaluated cardiovascular complications in hospitalized COVID‐19 patients and cardiovascular risk factors or comorbidities on mortality. The weighted summary proportion under the random‐effects model was computed using a Freeman Tukey transformation. Cochran *Q* test was applied to assess heterogeneity. The common mechanisms of COVID‐19 causing CVD complications have also been investigated by Bansal^22^ some of which are direct myocardial injury, systematic inflammation, and adverse effects of various therapies. As discussed by Bansal, the COVID‐19 virus binds to ACE 2, which leads to acute lung and myocardial injury. Moreover, COVID‐19 may cause systematic inflammation leading to multiorgan failure. Even the medicine used to treat COVID‐19 may have negative effect on the cardiovascular system.

Information on the risk factors for COVID‐19 mortality in low‐ and middle‐income countries is still rare. Martins‐Filho et al.^23^ scrutinized the factors associated with in‐hospital COVID‐19 death in Brazil. Mortality‐associated factors including age, gender, and preexisting comorbidities were identified by using logistic regression. Ciceri et al.^24^ exploited radiological and clinical risk factors at admission and predictors of clinical outcomes. Out of 410 hospitalized patients, 56.3% had comorbidities, with chronic kidney failure, diabetes, hypertension, and CAD. Li et al.^25^ studied a cohort of 507 nonsurvivors and 568 survivors from North America, European regions, and China with COVID‐19 mortality. Compared with nonsurvivors, survivors had more incidences of comorbidities such as COPD and cerebrovascular disease. Ciardullo et al.^26^ collected 373 adult patients' medical records on comorbid conditions, diabetes status, and laboratory findings. They focused on most prevailing comorbid conditions such as CVD, hypertension, and malignant neoplasms.

Small sample size and lack of taking many risk factors into consideration are some of the drawbacks of the studies reviewed above. To the best of our knowledge, we are the first taking into account the determinants like risk factors, symptoms, and comorbidities that led to mortality in CVD patients who are hospitalized with COVID‐19.

## 2 METHODS

We completed a questionnaire for each patient that included questions on symptoms such as fever, cough, myalgia, level of consciousness, chest pain, chills, abdominal pain, nausea, vomiting, diarrhea, and anorexia. The studied comorbidities consisted of diabetes, cancer, pulmonary diseases, renal diseases, hematologic disorders, neurological diseases, and hypertension. The risk factors of interest were sex, age, dialysis, smoking, addiction, and receiving immunosuppressive drugs. The items of the questionnaire were determined with the aid of medical experts in the fields of CVD and COVID‐19.

To collect data for our study, the medical reports of all CVD patients who have referred to Semnan hospital due to COVID‐19 infection were collected. This study was approved by the local ethical committee of Semnan hospital. All enrolled patients were informed about the aim of our study. All the patients completed written consent forms before their enrolment in the data collection procedure. Data collection was done by five nurses from January 2020 to January 2021. The nurses received four training sessions. The duration of each session was 2 h. The total number of collected samples in the aforementioned period was 660. The type of CVD of these patients was angina, myocardial infarction, stroke, heart failure, hypertensive heart disease, rheumatic heart disease, cardiomyopathy, abnormal heart rhythms, congenital heart disease, valvular heart disease, carditis, aortic aneurysms, peripheral artery disease, thromboembolic disease, and venous thrombosis.

### 2.1 Statistical analysis

The extracted data were analyzed using Matlab 2018a software. To determine the differences between the deceased and alive groups, Fisher's exact test^27^, ^28^ was applied on categorical data and Wilcoxon rank‐sum test^29^, ^30^ was used for continuous data. The significance level of these tests was set to *p* ≤ .05.

## 3 RESULTS

**Figure 1:**
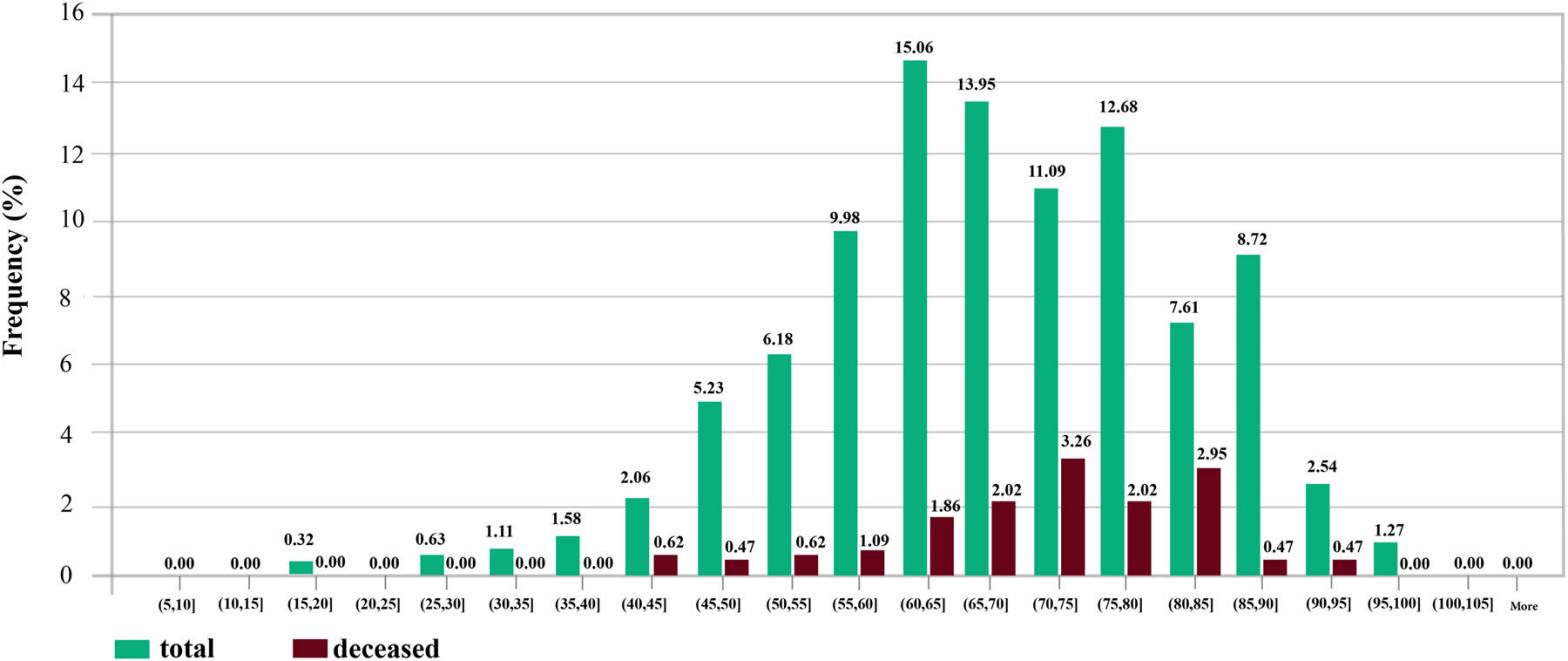
The relationship between the percentage of mortality rate and age of patients

The majority of patients (97.6%) had Iranian nationality. The percentage of male patients was 56.6%. The average and standard deviation of patients' age were 68 and 14 years. The youngest and oldest patients were 17 and 100 years old. To demonstrate the relation between mortality rate and patients' ages, in Figure 1, the age interval of the patients is divided into subintervals {(5,10),(10,15),…,(100,105]}. For each subinterval $\left(l_{i},h_{i}\right]$, two (green and red) bins are displayed. The green bin shows the percentage $\frac{100\times g_{i}}{660},$ where $g_{i}$ is the number of patients whose ages fall within subinterval $\left(l_{i},h_{i}\right]$ and 660 is the total number of patients in the data set. The red bin shows the percentage $\frac{100\times r_{i}}{660}$ where $r_{i}$ specifies the number of deceased patients whose ages fall within subinterval $\left(l_{i},h_{i}\right)$. For example, the age of 15.06% of the patients fell in the subinterval (60, 65) and 1.86% of the patients with age within (60, 65) were deceased.

The real‐time reverse transcriptase‐polymerase chain reaction (RT‐qPCR) is the molecular‐based assay used globally to detect SARS‐CoV‐2. The RT‐qPCR test was performed on 96.5% of the patients. Among the patients who referred to the hospital, 41.3% were admitted to the intensive care unit (due to their severe conditions), and the rest to the isolated and normal wards. All patients reported contact with a COVID‐19 infected person (about 12% had direct contact).

In the investigated patients, fever and myalgia were common symptoms. The average ± SD (i.e., standard deviation) of patients' body temperature at the time of referral was 37 ± 0.6°C and 22.6% of patients had fever at the time of referral. Totally, the average ± SD duration of hospitalization was 6.14 ± 6.6 days.

Of the investigated patients, 15.5% died and mortality was not significantly different between men and women (*p* = .515). Symptoms such as fever, cough, myalgia, seizures, anosmia, ageusia, dizziness, paresis, chest pain, weakness, dyspnea, chills, abdominal pain, nausea, vomiting, diarrhea, and anorexia were also occurred without affecting COVID‐19 mortality of CVD patients (*p* > .05). There was a significant difference between mortality and complaint of headache in cases with CVD who were infected with COVID‐19 (*p* = .000). LOC was also significantly associated with COVID‐19 mortality (*p* = .000). Oxygen saturation less than 93% and the need for mechanical ventilation were associated with COVID‐19 mortality since their *p* values were *p* = .000 and *p* = .000, respectively.

Addiction, smoking status, DM, underlying cancer, lung disease, asthma, kidney disease and dialysis, chronic hematological disease, neurological diseases, and hypertension had no association with COVID‐19 mortality (*p* > .05). The association between liver disease and COVID‐19 mortality was a borderline effect (*p* = .050). The presence of signs on computed tomography (CT) scan was significantly related to mortality in COVID‐19 cases (*p* = .004).

Among participants, 12% were in direct contact with an infected person, 18% of them were deceased, but no significant difference was observed between the patient's death in the setting of CVD and contact with an infected person (*p* = .309). No significant difference was observed between the patient's age and hospitalization length (*p* = .138), but there was a significant difference between mortality and patient's age (more than 80 years old, *p* < .001). There was a significant difference between mortality and hospitalization ward (isolated ward, critical care unit, general ward) (*p* < .001). There was a higher hospitalization length and mortality rate in cases hospitalized at critical care unit and isolated ward, respectively. Indeed, there was a significant difference between female gender and duration of hospitalization (with an average of 7.03 ± 6.97, *p* = .004). Thus, the duration of hospitalization in women was significantly longer than in men.

**Figure 2:**
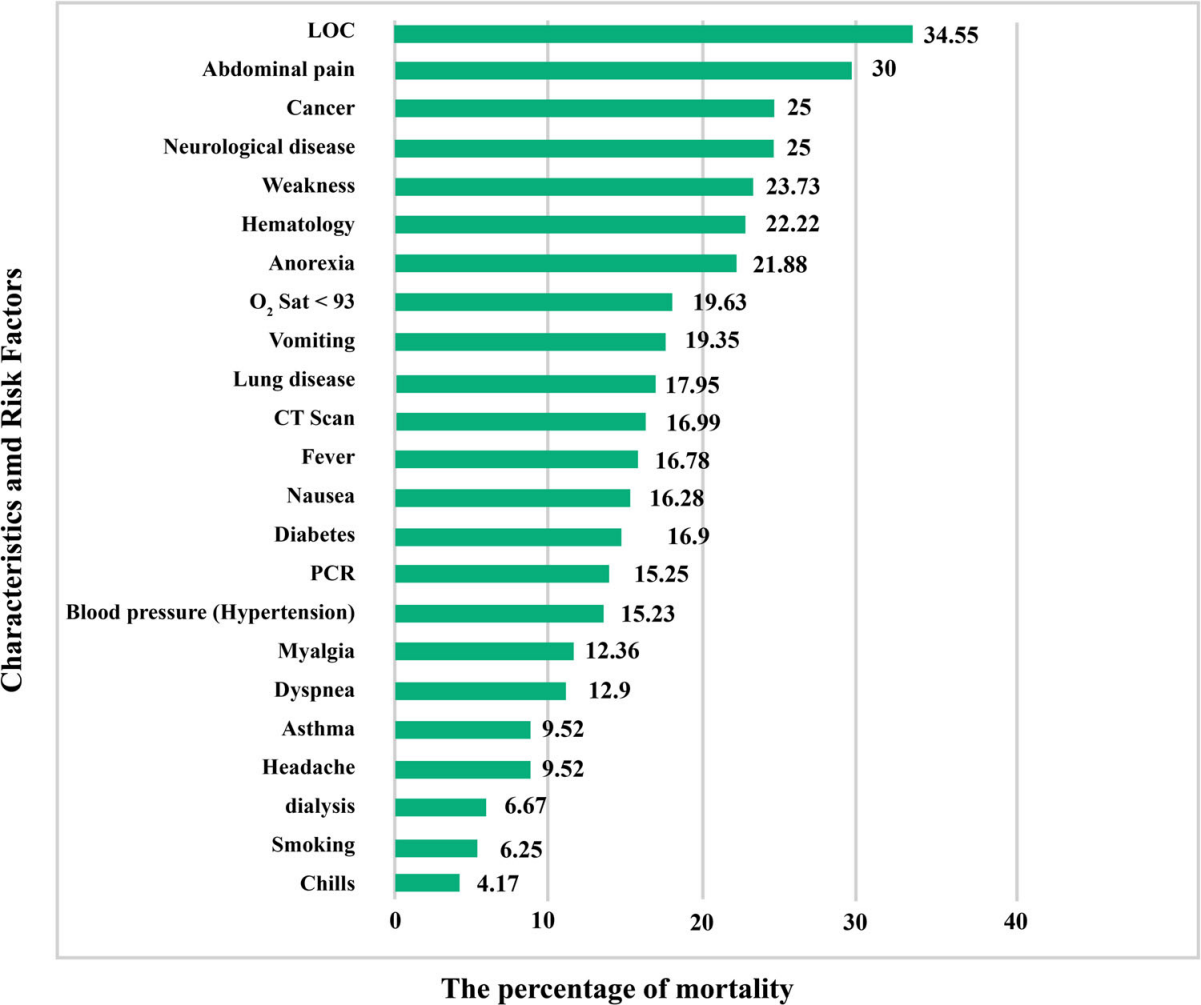
The mortality rate of COVID‐19 cardiovascular patients with specific characteristics, habits, and underlying diseases. LOC, loss of consciousness; PCR, polymerase chain reaction

**Figure 3:**
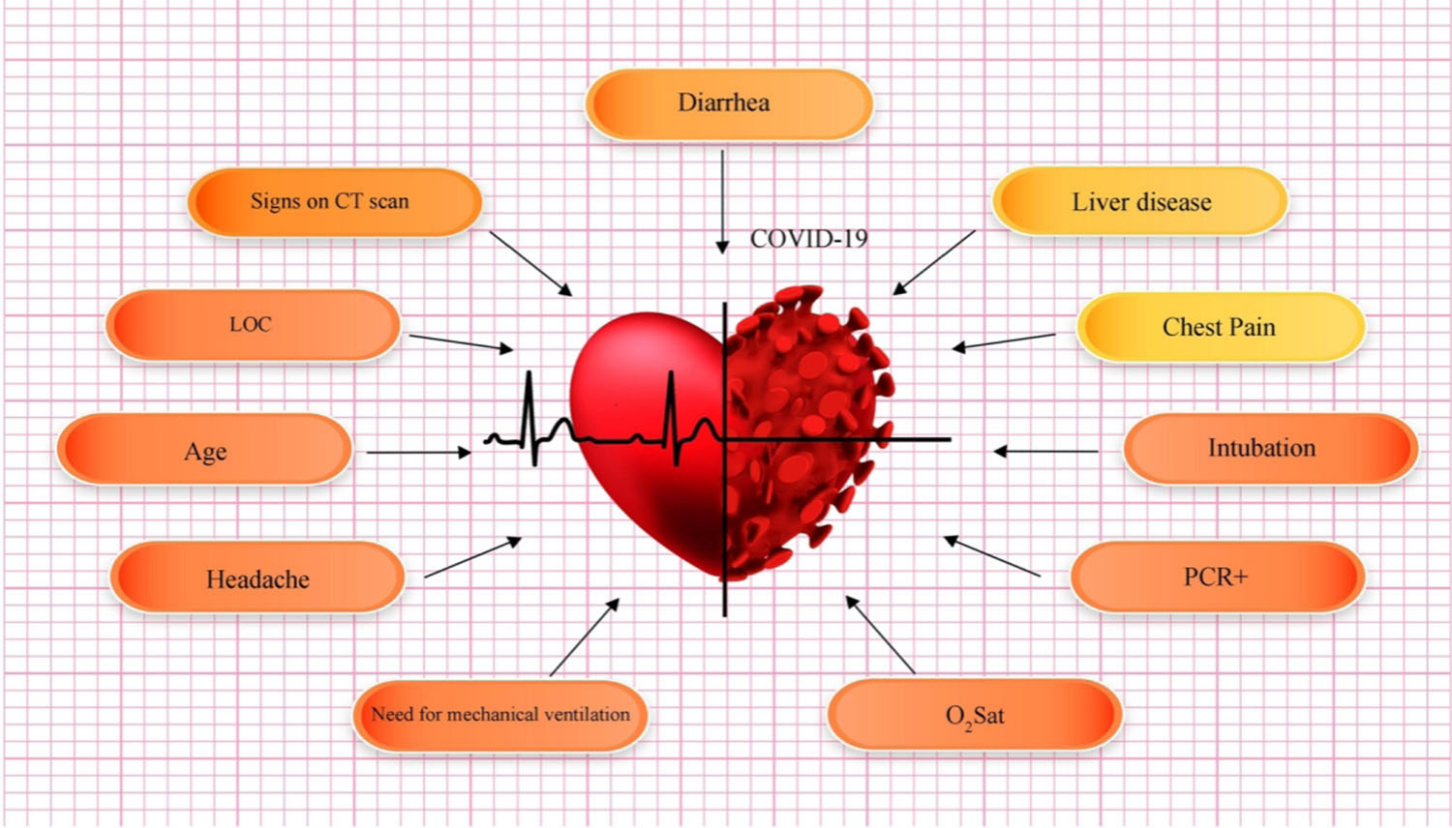
The most significant (*p* ≤ .05) risk factors, symptoms, and comorbidities in mortality rate of patients with cardiovascular disease and COVID‐19: darker colors represent more important factors (lower *p* value)

Statistical data of different risk factors in patients are listed in Table 1. Correlation between different symptoms and risk factors of COVID‐19 for CVD patients are listed in Tables 2 and 3, respectively. Figure 2 shows the relationship between specific characteristics, habits, and underlying diseases of cardiovascular patients and their COVID‐19 mortality rate. For example, as shown in Figures 2, 34.55% of patients with LOC died of COVID‐19. In Figure 3, the most significant risk factors, symptoms, and comorbidities in mortality rate of patients with CVD and COVID‐19 are illustrated. All of the items in Figure 3 are associated with *p* ≤ .05. The liver disease is the borderline effect with *p* = .05. Darker colors in Figure 3 represent the lower *p* value of their corresponding items.

**Table 1:** Demographics, clinical characteristics, and comorbidities of studied patients

Characteristics	Total (%)	Deceased (%)	Alive (%)	*p* value
Inpatient department				
Isolated	23.1	42.8	57.2	<.001
Critical care	41.3	10	90	
General ward	35.7	4.3	95.7	
Risk factors				
Sex				
Male	56.6	16.4	83.6	.515
Female	43.4	14.3	85.7	
Age category				
≤40	3.5	0	100	<.001
40–60	24	7	93	
61–80	52.7	15.3	84.7	
81–100	19.9	29	71	
Dialysis	2.3	6.7	93.3	.163
Smoking	2.4	6.25	93.75	.263
Addiction	3.5	13	87	.512
Receiving immunosuppressive drugs	0.2	0	100	>.999
Symptoms				
Fever	22.6	16.7	83.3	.27
Myalgia	13.5	12.4	87.6	.383
Seizures	0.3	0	100	>.999
Anosmia	0.8	0	100	.603
Ageusia	0.2	0	100	>.999
Dizziness	0.9	0	100	.598
Paresis	0.5	0	100	>.999
Skin lesions	0	0	0	NA
Weakness and lethargy	9	23.7	76.3	.068
Chills	3.6	4.2	95.8	.119
Abdominal pain	1.5	30	70	.198
Nausea	6.5	16.3	83.7	.868
Vomiting	4.7	19.3	80.7	.532
Diarrhea	1.5	40	60	.3
Anorexia	4.9	21.9	78.7	.298
Cough	24.7	15.3	84.7	.954
LOC	8.3	34.5	65.5	.000
Headache	3.2	9.5	90.5	.000
Chest pain	9.3	4.9	95.1	.14
Having symptoms on CT scan	86.6	17	83	.004
Need for mechanical ventilation	5.5	69	31	.000
Oxygen saturation less than 93%	66.6	21.2	78.8	.000
Comorbidity				
Other chronic diseases	8.3	21.8	78.2	.241
Kidney disease	7.7	51.1	84.9	.398
Neurological diseases	4.9	25	75	.133
Asthma	3.2	9.5	90.5	.449
Lung diseases	5.9	17.9	82.1	.661
Cancer	3	25	75	.239
Chemotherapy	0.8	20	80	>.999
Liver disease	0.5	33.3	66.7	.050
Diabetes	32.3	16.9	83.1	.485
Chronic hematological diseases	1.4	22.2	77.8	.576
Hypertension	40.2	17.4	96.6	.274

Abbreviations: CT, computed tomography; LOC, loss of consciousness.

**Table 2:** Correlation between different symptoms of cardiovascular disease patients hospitalized with COVID‐19 disease

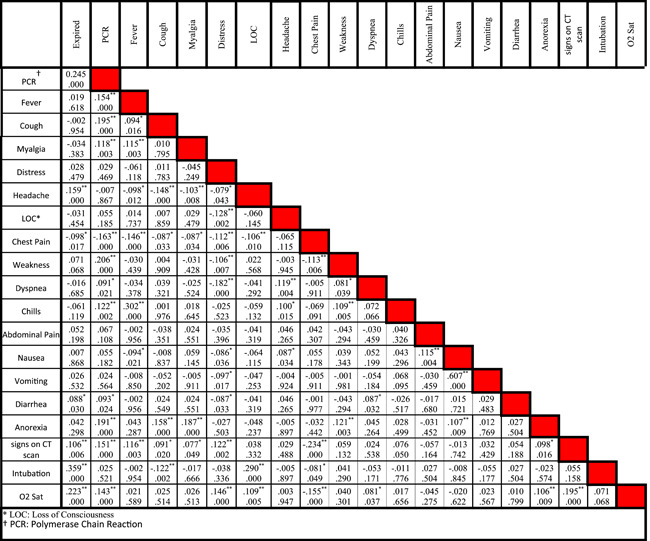

*Note*: In each cell, the number at the top is the correlation between two clinical characteristics while the number at the bottom is the p‐value.

Abbreviations: LOC, loss of consciousness; PCR, polymerase chain reaction.

* Means the correlation is significant at the 0.05 level (two‐tailed).

** Means the correlation is significant at the 0.01 level (two‐tailed).

**Table 3:** Correlation between different comorbidities in patients with COVID‐19 and cardiovascular disease. In each cell, the the number at the top is the correlation between two comorbidities while the number at the bottom is the p‐value

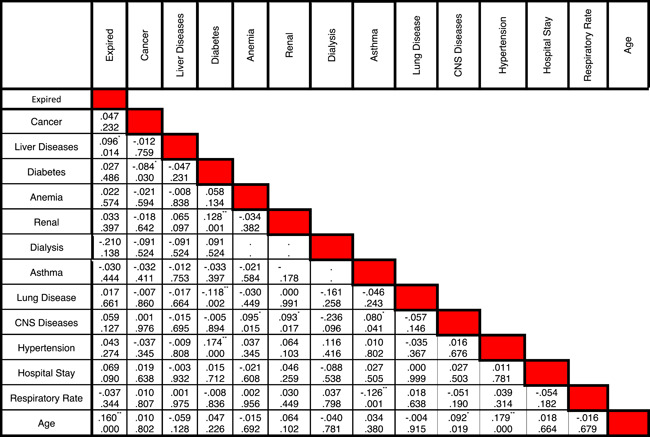

* Means the correlation is significant at the 0.05 level (two‐tailed).

** Means the correlation is significant at the 0.01 level (two‐tailed).

## 4 DISCUSSION

To the best of our knowledge, there are a few papers published about the relationships between different characteristics and risk factors of the COVID‐19 cardiovascular patients and their mortality rate. The correlation between different diseases in COVID‐19 patients with mortality was listed in Table 3.

Unlike existing research, in our study, the characteristic of cases with documented COVID‐19 infection and underlying CVDs and identification of risk factors associated with in‐hospital mortality were described. The main findings of our study were the significant association between age, headache, LOC, signs on CT scan, oxygen saturation less than 93%, need for mechanical ventilation, and hospitalization in the isolated ward with mortality in COVID‐19 cardiovascular patients. The female gender was associated with higher duration of hospitalization but had no impact on mortality in these cases. Among other comorbidities besides CVDs, other underlying diseases were not associated with COVID‐19 mortality, while liver disease showed a borderline effect in this regard.

Regarding the impact of age on mortality in cases with CVD, our data are in line with Li et al. who reported the cut point of age more than 65 years old.^8^ However, we observed no association between patient's age and hospitalization duration. We observed no relationship between mortality and gender in our cardiac cases, which is in contrast with Li et al. research who found higher mortality in their male participants.^8^ However, higher hospitalization length was observed in female cases with underlying cardiac problems. Higher hospitalization length in admitted cases to critical care unit and higher mortality rate in cases admitted in the isolated ward is also another finding of our cardiovascular cohort. It needs more evaluation to be included as a risk factor of mortality in cardiac cases due to scant data on pure cardiac cases infected with COVID‐19. To the best of our knowledge, this is the first study in this setting.

Regarding symptoms related to mortality in cases with CVDs, we observed no association between mortality and symptoms such as fever, chills, cough, myalgia, seizure, anosmia, ageusia, dizziness, paresis, chest pain, weakness, fatigue, abdominal pain, nausea, vomiting, diarrhea, and anorexia. On the other hand, headache, LOC, O_2_ saturation less than 93%, and need for mechanical ventilation were mortality‐related factors.

Regarding other preexisting comorbidities, we observed no relationship in cardiac cases between mortality and diabetes, cancer, pulmonary diseases, renal diseases, hematologic disorders, neurological diseases, and hypertension. The investigation performed by Li et al. on COVID‐19 cases with CVDs demonstrated the association between hypertension and mortality,^8^ which is inconsistent with our data that could be due to different populations of the study. Since the focus of COVID‐19 infection in lung tissue, we expected to observe the association between COVID‐19 mortality of CVD patients and lung diseases, but we did not observe such an association. The presence of diabetes is considered to be a predisposing factor for severe forms of COVID‐19 infection by Rastad et al.^5^

The prevalence of chronic kidney diseases is high in CVD patients, but interestingly we observed no COVID‐19 infected cases with concurrent CVD and renal disease. The absence of renal disease in our cardiovascular cohort seems to be odd. The borderline association between liver disease and COVID‐19‐related mortality in cases with CVD is also inconclusive and needs further evaluation due to the small number of cases with hepatic disease.

### 4.1 Limitations of our study

In this study, we did not include cases without CVDs to be compared versus cases with CVDs. Our study is based on samples collected from just one hospital so it is necessary to perform the study on more versatile samples from various medical centers in different regions. Another limitation of our work is the necessity to inspect the CVD patients' medical records to determine their CVD type confidently. During the pandemic, the hospital was already under pressure due to the shortage of medical experts. Assigning nurses to record data for our study amplified the personnel shortage. Therefore, we were forced to rely on a handful of nurses to record the data which slowed down the data collection process.

## 5 CONCLUSION

The findings of our study suggest advanced age, headache, need for the mechanical ventilator, low oxygen saturation, hospitalization in isolated wards, signs on CT scan, and LOC are factors associated with mortality in COVID‐19 infected cardiovascular patients. These factors might help physicians to determine high‐risk cases and take preventive actions.

## CONFLICT OF INTERESTS

The authors declare that there are no conflict of interests.

## ETHICS STATEMENT

The study was approved by the Semnan Hospital Ethics Committee.

### AUTHOR CONTRIBUTIONS

Roohallah Alizadehsani, Mohaddeseh Behjati, Azadeh Haddadi, Afshin Shoeibi, Fariba A. Sani, Navid H. Izadi, Zahra Roshanzamir, and Fahime Khozeimeh had continuation to prepare the first draft. Zahra A. Sani, Nizal Sarrafzadegan, Abbas Khosravi, Sheikh M. S. Islam, Mohaddeseh Behjati, and Saeid Nahavandi contributed to edit final draft. Roohallah Alizadehsani, Navid H. Izadi, Mehrdad Zahmatkesh, Rahimeh Eskandarian, Afshin Shoeibi, and Mohamad Roshanzamir contributed to all analysis of the data and produced the outcomes accordingly. Roohallah Alizadehsani, Afshin Shoeibi, Mehrdad Zahmatkesh, Rahimeh Eskandarian, Fariba A. Sani, Fahime Khozeimeh, and Mohamad Roshanzamir found the papers and then extracted data. Zahra A. Sani, Nizal Sarrafzadegan, Sheikh M. S. Islam, Abbas Khosravi, and Saeid Nahavandi provided overall guidance and managed the project.

### DATA AVAILABILITY STATEMENT

The data set is available from the corresponding author on request.
